# Effect of Different Types of Sequence Data on Palaeognath Phylogeny

**DOI:** 10.1093/gbe/evad092

**Published:** 2023-05-25

**Authors:** Naoko Takezaki

**Affiliations:** Laboratory of Life Sciences, Faculty of Medicine, Kagawa University, Kagawa, Japan

**Keywords:** coding sequence, noncoding sequence, outgroup, nucleotide composition, RY-coding, gene tree

## Abstract

Palaeognathae consists of five groups of extant species: flighted tinamous (1) and four flightless groups: kiwi (2), cassowaries and emu (3), rheas (4), and ostriches (5). Molecular studies supported the groupings of extinct moas with tinamous and elephant birds with kiwi as well as ostriches as the group that diverged first among the five groups. However, phylogenetic relationships among the five groups are still controversial. Previous studies showed extensive heterogeneity in estimated gene tree topologies from conserved nonexonic elements, introns, and ultraconserved elements. Using the noncoding loci together with protein-coding loci, this study investigated the factors that affected gene tree estimation error and the relationships among the five groups. Using closely related ostrich rather than distantly related chicken as the outgroup, concatenated and gene tree–based approaches supported rheas as the group that diverged first among groups (1)–(4). Whereas gene tree estimation error increased using loci with low sequence divergence and short length, topological bias in estimated trees occurred using loci with high sequence divergence and/or nucleotide composition bias and heterogeneity, which more occurred in trees estimated from coding loci than noncoding loci. Regarding the relationships of (1)–(4), the site patterns by parsimony criterion appeared less susceptible to the bias than tree construction assuming stationary time-homogeneous model and suggested the clustering of kiwi and cassowaries and emu the most likely with ∼40% support rather than the clustering of kiwi and rheas and that of kiwi and tinamous with 30% support each.

**Significance**

Different phylogenetic relationships are sometimes estimated by using different types of sequence data, whether coding or noncoding. Using different types of sequence data of palaeognaths, this study showed that 1) although the extent of estimated gene tree error tended to be high using loci with low divergence levels and/or short length, bias in estimated tree topology likely occurred with loci with high divergence and/or nucleotide composition bias and heterogeneity and that 2) the topological bias occurred more likely at coding loci than noncoding loci.

## Introduction

Palaeognathae is one of two groups of extant birds (Neornithes). The other group is Neognathae that comprises Galloanserae (chicken, duck, and geese) and Neoaves (the remaining birds) (Cracraft et al. 2004; van Tuinen and Dyke 2004; Mayr 2011; Jarvis et al. 2014; Brusatte et al. 2015; Claramunt and Cracraft 2015; Prum et al. 2015). Extant palaeognaths consist of five groups: (1) flighted Tinamiformes (tinamous) and four groups of flightless palaeognaths (ratites): (2) Struthioniformes (ostriches), (3) Rheiformes (rheas), (4) Casuariiformes (cassowaries and emus), and (5) Apterygiformes (kiwi). Morphological studies supported the relationships of two extinct lineages, Dinornithiformes (moas) and Aepyornithidae (elephant birds), with other palaeognaths variably (Livezey and Zusi 2007; Worthy and Scofield 2012). However, molecular studies supported the grouping of moas with tinamous (Phillips et al. 2010; Haddrath and Baker 2012; Baker et al. 2014; Cloutier et al. 2019; Sackton et al. 2019) and elephant birds with kiwi (Mitchell et al. 2014; Grealy et al. 2017; Yonezawa et al. 2017).

**Fig. 1.**
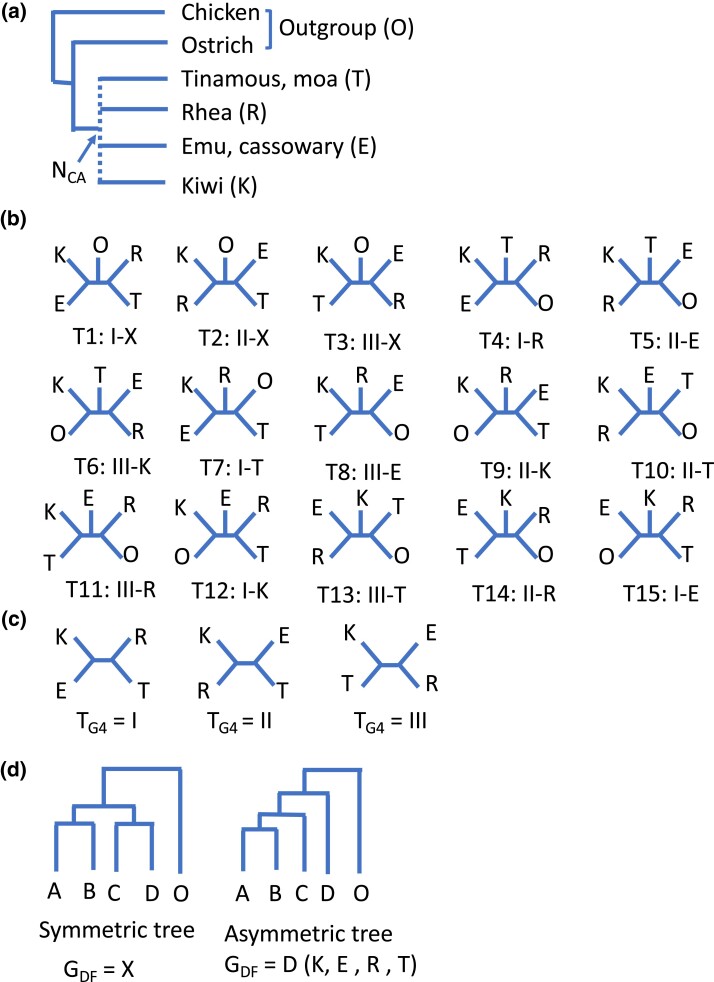
The phylogenetic relationships of palaeognaths. O, outgroup, ostrich and chicken; T, tinamous and moa; R, rheas; E, emu and cassowary; K, kiwi. (*a*) The relationships of the palaeognath birds investigated in this study. Ostrich was regarded as the outgroup in addition to chicken. *N*_CA_, the common ancestral node of the four palaeognath groups (Tinamous, Rhea, Emu, and Kiwi) represented by T, R, E, and K. (*b*) Fifteen possible relationships of the four groups and the outgroup (T1–T15). (*c*) The three possible relationships of the four groups (T, R, E, and K) (T_G4_): I–III. (*d*) Two types of the branching pattern of the four palaeognath groups regarding the group that diverged first among them (*G*_DF_). In the asymmetric tree, D diverged among the four groups A, B, C, and D, which represent each of the four palaeognath groups (K, E, R, and T). In the symmetric tree, the four groups split into two clades consisting of two groups each, and *G*_DF_ is denoted as X. Previous studies that constructed phylogeny of the five groups are as follows: Slack et al. 2007 (mtgenome, T7); Hackett et al. 2008 (19 nuclear loci, T4); Harshman et al. 2008 (20 nuclear loci, T4 and T1); Phillips et al. 2010 (mitogenome with third codon positions RY-coded, T4); Haddrath and Baker 2012 (27 nuclear genes, T7; 10 nuclear genes, T13); Mitchell et al. 2014 (mitogenome with third codon positions RY-coded, T4); Prum et al. 2015 (256 nuclear loci, T14); Grealy et al. 2017 (mtgenome with third codon positions with RY-coding and 154 nuclear protein-coding genes, T4; nuclear genes only, T14); Reddy et al. 2017 (54 nuclear noncoding loci, T7); and Yonezawa et al. 2017 (mtgenome and 47 nuclear loci, T4). Sackton et al. 2019 (12,676 CNEEs, 5,016 introns, and 3,158 UCE loci, T7 by gene tree–based approach; concatenated introns and TENT [CNEEs, introns, and, UCEs], T4; concatenated CNEEs and UCEs, T14); Cloutier et al. 2019 (12,676 CNEEs, 5,016 introns, and 3,158 UCEs, T7 in gene tree–based approach and concatenated CNEEs and TENT, T14; concatenated introns and UCEs, T4); Braun and Kimball 2021 (256 nuclear loci from Prum et al. 2015, T14; using only noncoding loci, T13); Kuhl et al. 2021 (3′-UTR from transcriptomes, 7.9 ± 3.6 Mb alignment length, T14).

Tinamous are placed as a group that diverged first in the palaeognath phylogeny and separated from the ratites by most morphological studies (Cracraft 1974; Bledsoe 1988; Lee et al. 1997; Dyke and van Tuinen 2004; Leonard et al. 2005; Livezey and Zusi 2007; Bourdon et al. 2009; Worthy and Scofield 2012) (but see Elanowski 1995; Johnson 2011) and earlier molecular studies (Prager et al. 1976; Lee et al. 1997; García-Moreno and Mindell 2000; van Tuinen et al. 2000; Cooper et al. 2001; Braun and Kimball 2002; García-Moreno et al. 2003; Gibb et al. 2007; Slack et al. 2007; Le Duc et al. 2015). However, recent studies using a large number of genes and/or species support that ostriches diverged first, and the root was placed between the ostriches and other palaeognaths (Chojnowski et al. 2008; Hackett et al. 2008; Harshman et al. 2008; Phillips et al. 2010; Haddrath and Baker 2012; Kimball et al. 2013; Smith et al. 2013; Baker et al. 2014; Mitchell et al. 2014; Prum et al. 2015; Grealy et al. 2017; Reddy et al. 2017; Yonezawa et al. 2017; Cloutier et al. 2019; Sackton et al. 2019; Braun and Kimball 2021; Kuhl et al. 2021). In contrast, the relationships among the five groups (fig. 1) are variably supported (T4, T7, T13, and T14; see more details for the legend of fig. 1) and often accompanied by low statistical support (e.g., Braun et al. 2019).

The difficulty of resolving the phylogenetic relationships among the groups within palaeognaths is presumably due to short intervals of lineage divergences and rate variation among the lineages (Cracraft et al. 2004). When divergences of multiple lineages occur in short intervals, the phylogenetic relationships at each locus (gene trees) can be different from locus to locus by incomplete lineage sorting (ILS), which occurs in the coalescent process of alleles in the ancestral population and may not be congruent with the phylogenetic relationships of species (e.g., Haddrath and Baker 2012; Xu and Yang 2016). The phylogenetic trees have been constructed from concatenated sequence of multiple loci to reduce the effect of sampling error particularly to resolve the divergence patterns within short intervals (Philippe et al. 2005, 2017; Gatesy and Springer 2014). However, the concatenation approach can be inconsistent in extreme cases of ILS (Kubatko and Degnan 2007; Degnan and Rosenberg 2009; Roch and Steel 2015; Mendes and Hahn 2018). In the presence of ILS, species trees can be estimated by approaches that take the coalescent process into account (e.g., Rannala and Yang 2003; Liu et al. 2010; Mirarab et al. 2014a). Summary gene tree methods, in which gene trees are estimated first, are commonly used, because the methods in which gene trees and species tree are estimated simultaneously (e.g., Heled and Drummond 2010; Dasarathy et al. 2015; Flouri et al. 2022) are computationally demanding. However, summary gene tree–based methods can perform poorly when estimation errors are present in gene trees (DeGiorgio and Degnan 2010; Leaché and Rannala 2011; Bayzid and Warnow 2013; Lanier et al. 2014; Mirarab et al. 2014b; Bayzid et al. 2015). Moreover, estimated gene trees are error-prone due to the limited sequence length. The existence of recombination breakpoint(s) within a locus can also cause error in the estimated gene tree. In addition, unbalanced sampling of loci with different underlying gene trees due to ILS can cause error in species tree estimation. The neutrality of sequence evolution that the multispecies coalescent methods assume may not hold in actual data (e.g., Gatesy and Springer 2014; Springer and Gatesy 2016). Cases in which extensive heterogeneity of gene trees was caused by estimation error in actual data were reported (Scornavacca and Galier 2017; Richards et al. 2018).

In the case of the palaeognath phylogeny, extensive heterogeneity of topologies was observed among estimated gene trees (Cloutier et al. 2019; Sackton et al. 2019). However, the extent of error in gene tree estimation and its effect on the species tree estimation are unknown. The accuracy of phylogenetic trees can be affected by many factors such as species included, substitution model used, sequence length, the extent of sequence divergence, long branch attraction (Philippe et al. 2005), and data type (e.g., coding vs. noncoding, Chen et al. 2017; Reddy et al. 2017). These factors can create not only the sampling errors but also bias in gene tree estimation.

In this study, the extent and the effect of gene tree estimation error on the phylogenetic relationships of palaeognaths and the factors that affect gene tree estimation error and the phylogenetic relationships of the palaeognath groups were investigated using sequence data from Cloutier et al. (2019) (12,676 conserved nonexonic element [CNEE] loci, 5,016 intron loci, and 3,158 ultraconserved element [UCE] loci) and Sackton et al. (2019) (8,721 protein-coding loci) (tables 1 and S1, Supplementary Material online).

**Table 1:** Data Sets Used in This Study

Data Set	Codon Position	No. of Loci	No. of Species		No. of Sites		No. of Parsimony Informative Sites		Missp	
			All	Average	Total	Average	Total	Average	Total	Average
CNEE		12,561	15	14.9 ± 0.3	4,734,794	376.9 ± 146.4	257,037	20.5 ± 14.6	0.03	0.02 ± 0.03
Intron		1,802	15	14.6 ± 0.5	4,335,265	2,405.8 ± 2,736.7	947,861	526.0 ± 547.3	0.23	0.19 ± 0.07
UCE		1,363	15	14.7 ± 0.5	3,434,146	2,519.5 ± 456.3	476,999	369.5 ± 129.7	0.16	0.13 ± 0.09
CDS	C123	5,374	14	13.7 ± 0.7	11,160,875	2,076.8 ± 1,500.9	1,041,063	193.7 ± 163.3	0.16	0.15 ± 0.11
	C12	5,374	14	13.7 ± 0.7	7,440,627	1,384.6 ± 1,000.6	320,877	59.7 ± 71.2	0.16	0.15 ± 0.11
	C3	5,374	14	13.7 ± 0.7	3,720,248	692.3 ± 500.3	720,186	134.0 ± 104.4	0.16	0.15 ± 0.11

Note.—CNEE, intron, and UCE data are from Cloutier et al. (2019). CDS data are from Sackton et al. (2019). Loci in which there were unusually long branches for some species and recombination detected among the five groups (Kiwi, Emu, Rhea, Tinamou, and outgroup) were excluded.

Missp, proportion of missing data.

## Results

Using the data of CNEEs, introns, and UCEs from Cloutier et al. (2019) and protein-coding sequences (CDSs) from Sackton et al. (2019), four data sets (CNEE, intron, UCE, and CDS) were created. In these data sets, loci were excluded when there were unusually long branches, and recombination was detected among the five groups (kiwi [Kiwi], emu and cassowary [Emu], rheas [Rhea], tinamous and moa [Tinamou], and outgroups [ostrich and chicken]) and, additionally for CDSs, when positive selection was detected. CDS (C123, all codon positions) was divided into C12 (first and second codon positions) and C3 (third codon positions) (see Materials and Methods).

### Phylogenetic Trees Constructed From Concatenated Sequences

**Fig. 2.**
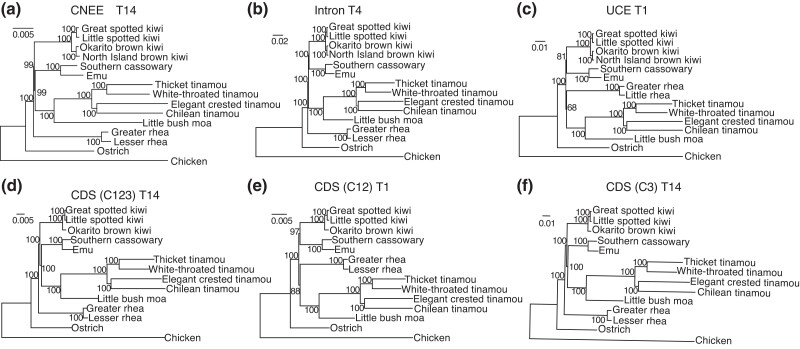
Phylogenetic trees from concatenated sequences. The trees were constructed partitioning with -p option of IQTREE by assuming GTR + G4 for each locus. Norecomb sets were used. The numbers at nodes are UFBoot values with 1,000 replications. The data sets used and tree topologies are (*a*) CNEE, T14; (*b*) intron, T4; (*c*) UCE, T1; (*d*) CDS (C123, all codon positions), T14; (*e*) CDS (C12, first and second codon positions), T1; and (*f*) CDS (C3, third codon positions), T14.

**Fig. 3.**
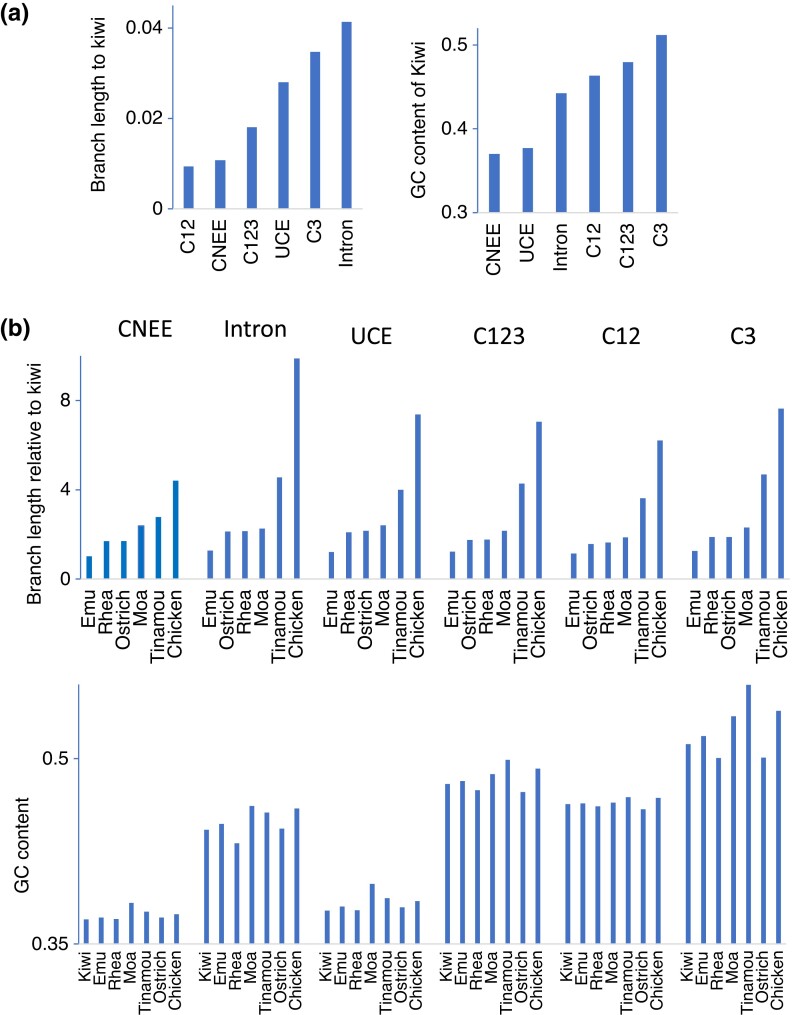
The comparison of sequence divergence level and GC content among different data sets and palaeognath groups. (*a*) The sequence divergence and GC content of different data sets. The average branch lengths from the common ancestral node of palaeognaths excluding outgroup (*N*_CA_ in fig. 1*a*) and GC content of Kiwi are shown. (*b*) The relative sequence divergence level and GC content of different species or species groups. The average branch lengths from the common ancestral node of palaeognaths relative to those of Kiwi and GC content of species or species groups are shown.

Figure 2 shows phylogenetic trees constructed from concatenated sequences of the different data sets. These trees included two outgroups, ostrich and chicken. Phylogenetic trees were also constructed using only ostrich or chicken as the outgroup (table 2). This is because distantly related outgroups can distort constructed tree topologies (e.g., Philippe et al. 2005, 2009; Takezaki and Nishihara 2016, 2017; Takezaki 2021). The sequence divergence of chicken was much higher (2.6–4.6 times) than that of ostrich (figs. 2 and 3*b* and supplementary table S2, Supplementary Material online).

**Table 2:** Phylogenetic Trees Constructed Using Different Outgroups by Concatenated and Gene Tree–Based Approaches

Data Set	Outgroup																													
	Chicken + Ostrich										Ostrich										Chicken									
	Concatenated					Gene Tree–Based					Concatenated					Gene Tree–Based					Concatenated					Gene Tree–Based				
	Tree			UFBoot		Tree			BP		Tree			UFBoot		Tree			BP		Tree			UFBoot		Tree			BP	
	A	G	D			A	G	D			A	G	D			A	G	D			A	G	D			A	G	D		
CNsEE	14	II	R	*	99	4	I	X	*	9.8	14	II	R	*	100	4	I	R	*	19	7	I	T		100	4	I	R		16
Intron	4	I	R	*	100	3	III	X	*	17	4	I	R	*	100	14	II	R	*	25	7	I	T		96	13	III	T	*	21
UCE	1	I	X	*	68	1	I	X	*	18	14	II	R	*	56	14	II	R	*	24	7	I	T		100	13	III	T	*	19
CDS																														
C123	14	II	R	*	100	4	I	R	*	14	14	II	R	*	100	14	II	R	*	25	10	II	T	*	100	7	I	T		21
C12	1	I	X	*	88	7	I	T		10.8	14	II	R	*	100	4	I	R	*	18.2	7	I	T		89	7	I	T		17.3
C3	14	II	R	*	100	4	I	R	*	14.6	14	II	R	*	100	14	II	R	*	24.3	10	II	T	*	98	7	I	T		20.7

Note.—Norecomb sets were used. Phylogenetic trees from concatenated sequences were constructed by partitioning with -p option of IQTREE. GTRG4 was used for each partition. Under “Tree,” A, G, and D represent tree number among the 15 possible tree topologies for the five groups (K, E, R, T, and outgroup), tree number among the three possible trees for the four groups (K, E, R, and T), and the group that diverged first among the four groups, respectively (see fig. 1*b–d*).

Fifteen possible tree topologies of the five groups (Kiwi, Emu, Rhea, Tinamou, and the outgroup) (T1–T15; fig. 1*b*) can be denoted by a combination of the relationships of the four palaeognath groups (*T*_G4_: I–III; fig. 1*c*) and the group that diverged first among them (*G*_DF_: Kiwi, Emu, Rhea, Tinamou, or X; fig. 1*d*). The asymmetric and symmetric trees shown in figure 1*d* are two types of the branching patterns with respect to the group that diverged first among the four groups, disregarding the branch lengths. In the asymmetric tree, D diverged first among the four groups A, B, C, and D, and D can be any one of the four groups. Because in the symmetric tree the four groups split into two clades consisting of two groups each, *G*_DF_ is denoted as X (fig. 1*d*). This notation clarified that the outgroup used strongly affected the constructed tree topologies (table 2). In the trees constructed with only ostrich as the outgroup, *G*_DF_ was Rhea for all the data sets, whereas *G*_DF_ was all Tinamou with only chicken as the outgroup. With both ostrich and chicken as the outgroup, *G*_DF_ was Rhea except for UCE. *T*_G4_ was either I or II, but compared with *G*_DF_, *T*_G4_ showed a less distinctive pattern depending on the outgroup and data sets used.

### Phylogenetic Trees Constructed by the Gene Tree–Based Approach

In the trees constructed by the gene tree–approach, except for CNEE, *G*_DF_ was Rhea or Tinamou when only ostrich or chicken was used as the outgroup, coinciding with those by the concatenated approach (table 2). In contrast to *G*_DF_, *T*_G4_ did not match those by the concatenated approach in most of the cases and trees I–III were all supported.

### Computer Simulation Using Different Outgroups

**Fig. 4.**
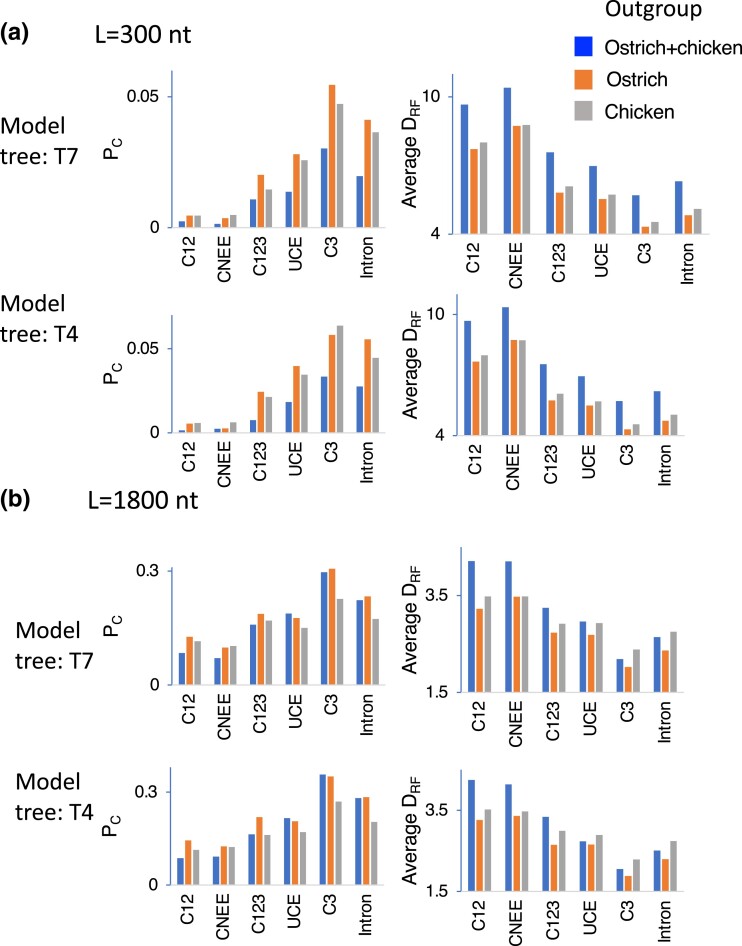
*P*_C_ and *D*_RF_ values for different outgroups in computer simulation. The cases in which the model tree was T7 and T4 and the sequence length (*L*) was (a) 300 nt and (b)1,800 nt are shown.

A computer simulation was carried out to see the effect of different outgroups on the constructed tree topology. The proportions of replications in which the correct tree topology (model tree) was constructed (*P*_C_s) and Robinson–Foulds distances (*D*_RF_s, twice the number of internal branches that differ from those of the correct tree topology) were calculated for the cases of the sequence lengths (*L*) 300, 600, 900, and 1,800 nt using (1) both chicken and ostrich and either (2) ostrich or (3) chicken as the outgroup for all the possible tree topologies of the five groups (T1–T15 in fig. 1*b*) by estimating branch lengths for concatenated sequences of all the data sets (supplementary table S3, Supplementary Material online). *P*_C_s and *D*_RF_s, when model trees were T4 and T7, and *L* was 300 and 1,800 nt, are shown in figure 4.

Even though values of *P*_C_ were small (0–0.09, 0.004–0.18, 0.02–0.23, and 0.05–0.44 when *L* was 300, 600, 900, and 1,800 nt, respectively), particularly for short sequence lengths and data sets with low sequence divergence such as CNEE and C12, *P*_C_s with (2) only ostrich as the outgroup were generally higher than those with (3) only chicken and with (1) both chicken and ostrich. The extent of topological error indicated by average *D*_RF_s was smaller (2) with ostrich than (1) with both chicken and ostrich or (3) with chicken as the outgroup except for a few cases. Because the cases in which *P*_C_s or *D*_RF_s with (2) ostrich as the outgroup were not higher or smaller than those with the other outgroups ((1) and (3)) tended to be observed when the sequence length was short and the sequence divergence was low, it is likely that these exceptional cases occurred due to stochastic error, though 1,000 replications were carried out when *L* was 600–1,800 nt and 5,000 or 10,000 replications when *L* was 300 nt. Therefore, the results of the computer simulation indicate that in terms of the sequence divergence level, the probability that the correct tree topology is constructed is higher by using (2) only ostrich as the outgroup than those using (3) only chicken and (1) both ostrich and chicken.

### The Group That Diverged First Among Kiwi, Emu, Rhea, and Tinamou

The above results by the concatenated and gene tree–based approaches showed that the outgroups strongly affected *G*_DF_ in the trees constructed. *G*_DF_ was Rhea with ostrich as the outgroup and Tinamou with chicken as the outgroup except for CNEE for which *G*_DF_ was Rhea even only with chicken as the outgroup, consistent with the result of Simmons et al. (2022). Together with the result of the computer simulation indicating that the correct tree topologies are more likely constructed using the closely related ostrich as the outgroup than the distantly related chicken and both ostrich and chicken, the results support that Rhea diverged first among the four palaeognath groups.

### Site Concordance Factors and Gene Concordance Factors

Although *G*_DF_ was strongly affected by the outgroup and the above result suggested that *G*_DF_ is Rhea, the relationship of the four palaeognath groups (*T*_G4_: I–III) (fig. 1*c*) varied depending on the data set, outgroup, and whether the concatenated or gene tree–based approach was used. To see the extent of concordance of the constructed phylogenetic trees and signals contained in sequence data for the four group trees, the gene concordance factor (gCF, frequency of the gene trees that contain the internal branch) and site concordance factor (sCF, average frequency of sites that support the internal branch in four sequences randomly chosen) by parsimony criterion (Minh et al. 2020b) were computed for internal branches of the three possible trees I–III of the four groups (table 3).

**Table 3:** gCFs and sCFs of Different Data Sets

Data Set	No. of Loci	Tree (*T*_G4_)	gCF	gCF_N	Concatenated Sequence		Average Per Locus^a^	
					sCF	sCF_N	sCF	sCF_N
	12,561	I	26.7	3,349	39.1	5,326.9	23.4 ± 27.9	0.43 ± 0.67
		II	24.6	3,095	31.5	4,284.7	18.8 ± 26.1	0.35 ± 0.59
		III	23.0	2,883	29.3	3,967.2	18.0 ± 25.6	0.32 ± 0.51
		Total	74.3	9,327	100.0		60.2	
	1,802	I	33.0	595	40.6	20,598.2	40.8 ± 10.7	12.90 ± 13.73
		II	33.6	606	30.5	15,209.3	30.4 ± 9.9	9.64 ± 10.47
		III	32.9	592	29.0	14,456.1	28.7 ± 9.7	9.14 ± 9.97
		Total	99.5	1,793	100.0		99.9	
	1,363	I	38.4	524	41.0	11,808.2	41.0 ± 10.2	9.55 ± 4.48
		II	30.6	417	30.4	8,726.0	30.7 ± 10.7	7.46 ± 6.15
		III	30.7	418	28.6	8,249.6	28.6 ± 9.0	6.63 ± 3.41
		Total	99.7	1,359	100.0		100.3	
C123	5,374	I	32.8	1,762	38.1	30,027.3	38.5 ± 13.7	5.95 ± 5.37
		II	35.1	1,887	33.2	26,132.4	33.1 ± 14.3	5.37 ± 5.35
		III	29.8	1,602	28.7	22,451.1	28.6 ± 12.6	4.45 ± 4.20
		Total	97.7	5,251	100.0		100.1	
C12	5,374	I	29.4	1,579	38.3	7,681.7	31.0 ± 25.2	4.00 ± 5.17
		II	28.0	1,507	32.9	6,535.0	26.6 ± 24.4	4.04 ± 5.19
		III	26.0	1,399	28.8	5,729.5	22.6 ± 22.2	4.00 ± 5.18
		Total	83.5	4,485	100.0		80.2	
C3	5,374	I	31.5	1,692	38.0	22,434.5	38.3 ± 15.0	11.6 ± 9.85
		II	34.5	1,856	33.4	19,484.8	32.9 ± 15.6	11.7 ± 9.87
		III	29.9	1,606	28.7	16,696.1	28.5 ± 14.0	11.6 ± 9.85
		Total	95.9	5,154	100.1		99.6	
C12 and	5,374	I	32.4	1,736	38.5	10,859.5	29.0 ± 27.2	0.90 ± 1.20
RY-coded		II	31.4	1,687	33.3	9,349.9	25.0 ± 26.6	0.86 ± 1.45
C3		III	28.1	1,510	28.2	7,874.9	19.5 ± 24.0	0.61 ± 0.91
		Total	91.9		100.0		73.4	
Intron	1,802	I	35.4	637	43.1	4,962.8	40.8 ± 10.7	12.90 ± 13.73
(RY-		II	31.0	558	29.4	3,400.4	30.4 ± 9.9	9.64 ± 10.47
coded)		III	32.0	576	27.5	3,160.3	28.7 ± 9.7	9.14 ± 9.97
		Total	98.3		100.0		99.9	

Note.—Trees I–III are three possible tree topologies of four groups (K, E, R, and T).

gCF, the proportion of loci in which the internal branch of the tree appeared in percent; gCF_N, the number of loci in which the internal branch of the tree appeared; sCF, the proportion of sites that support the internal branch of the tree in percent; sCF_N, the number of sites that support the internal branch of the tree.

a Average of the loci where the internal branch of tree I, II, or III appeared.

sCF that supports tree I was the highest among trees I–III in concatenated sequence and locus average of all the data sets. sCF was ∼40% for tree I and ∼30% for both trees II and III in the concatenated sequences of all the data sets and locus averages except for CNEE and C12. Locus averages of sCFs for CNEE and C12 were smaller than those for concatenated sequences and added up to only about 60% and 80%, respectively. Locus averages of SCF_Ns (the numbers of sites that support the internal branch) of trees I–III for CNEE and C12 (0.3–0.4 and 4) were smaller than the other data sets (4.5 or higher). Moreover, sCF_Ns that support trees I–III were all zero in 1,480 loci (12%) of CNEE and 400 loci (7%) of C12, although there were almost no such loci in the other data sets. This shortage of phylogenetic signal in individual loci in CNEE and C12 was likely due to their low sequence divergence (fig. 3) and the short sequence length of CNEE (377 bp on average) compared with those in the other data sets (1,385–2,520 bp) (table 1).

Correspondingly to sCFs, gCFs that support tree I were the highest in CNEE, UCE, and C12. However, gCFs that support tree II were the highest in intron, C123, and C3, although there was only a slight difference in values of gCF for trees I–III (32.9–33.6%) for intron. In addition, the divergence level of intron, C123, and C3 was higher than the other data sets, and in these data sets, GC content and the variation among the palaeognath groups tended to be higher than the other data sets (fig. 3). This suggests that tree II tended to be constructed more often in sequences with high divergence, GC content, and the heterogeneity. In previous studies, purine–pyrimidine (RY)-coding was used for the third codon positions in mitochondrial (Phillips et al. 2010; Grealy et al. 2017) and nuclear data (Nabholz et al. 2011) to alleviate the effect of multiple hits and heterogeneous nucleotide composition among species. Following these studies, using RY-coding for intron and third codon positions of C123, gCF became the highest for tree I, corresponding to sCF (table 3).

### Effect of Sequence Divergence Level on gCF and sCF and Nucleotide Composition Bias and Heterogeneity

The above results suggested that tree II tended to be supported more often due to multiple hits in high divergence loci and possibly the complex substitution pattern with high and/or heterogeneous GC content among lineages. To confirm this idea, by creating subsets of loci with top-10% to −90% of high divergence, the concordance pattern of gCFs and sCFs for trees I–III and the relationship with bias of nucleotide composition and its heterogeneity among lineages were investigated.

**Fig. 5.**
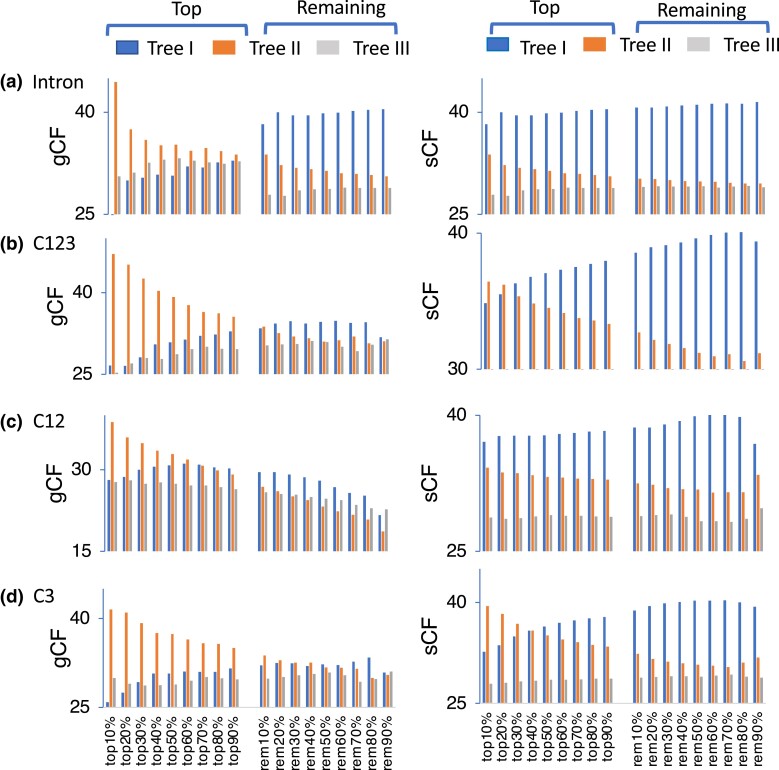
gCFs and sCFs supporting trees I–III of the four palaeognath groups in top and rem locus sets of different sequence divergence levels. Top-10% to −90% locus sets of high divergence were created by sorting the loci with average branch lengths from the common ancestral node of the four palaeognath groups (Kiwi, Emu, Rhea, and Tinamou). Rem-10% to −90% locus sets are created by excluding the corresponding top locus sets. gCFs and sCFs of data sets (a) intron, (b) C123, (c) C12, and (d) C3 are shown. The values of all the data sets are shown in supplementary fig. S1, Supplementary material online.

In the analysis of the locus sets, as the sequence divergence increased, gCF that supports tree II became higher in all the data sets except for UCE. sCF that supports tree II also generally showed slight increase with high sequence divergence (supplementary fig. S1 and table S4, Supplementary Material online). gCF that supports tree II was the highest among those for trees I–III in all top locus sets of intron, C123, C3, and, unexpectedly, up to top-60% locus sets of C12, which has low sequence divergence (fig. 5). In these data sets, however, when up to top-40% of high divergence loci were excluded, in the remaining loci (rem locus sets), gCF that supports tree I became the highest. In the case of C123 and C3, even sCF that supports tree II was the highest in up to the top-40% locus sets. Similar to gCF, by excluding the top-10% of high divergence loci, sCF that supports tree I became the highest (fig. 5).

Similar results were obtained by computing gCFs and sCFs for top-10% to −90% locus sets of high GC content (supplementary fig. S2 and table S5, Supplementary Material online). Because GC content and sequence divergence are positively correlated (Pearson's correlation coefficient is 0.19–0.58 for different data sets) (supplementary table S6, Supplementary Material online), the effect of these factors cannot be separated. However, the effect of GC content on the estimated gene trees appears to be slightly stronger than that of sequence divergence. sCF that supported tree II became the highest among those for trees I–III in more top locus sets of C123 (up to top 40%) and C12 (up to top-20%) than in top locus sets of high sequence divergence. In some of the top locus sets (top-90% of UCE, top-30%, −50% to −90% of C12, and top-20% to −60% of C3), even gCF that supports tree III became the highest.

**Fig. 6.**
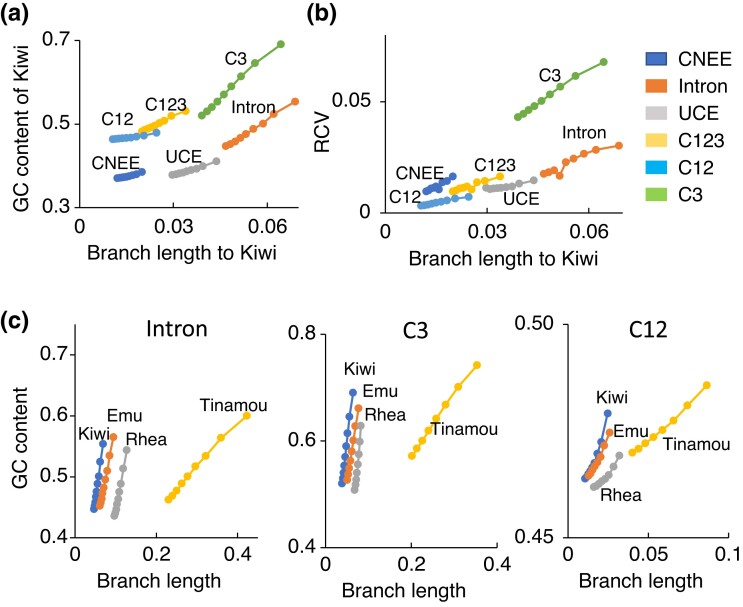
Relationships between sequence divergence and GC content as well as RCV in top-10% to −90% locus sets of different data sets and the four palaeognath groups. (*a*) Relationship between sequence divergence and GC content. (*b*) Relationship between sequence divergence and RCV. In (*a*) and (*b*), sequence divergence is represented by branch lengths to Kiwi. (*c*) Relationships between sequence divergence and GC content in intron, C3, and C12. Supplementary fig. S3, Supplementary Material online, shows those for all the data sets.

Figure 6 shows that bias (GC content) of nucleotide composition in each species group (see also supplementary fig. S3, Supplementary Material online) and heterogeneity of nucleotide composition (relative compositional variability [RCV], Phillips and Pratt 2008, see Materials and Methods) among the four palaeognath groups increased as sequence divergence increased. Particularly for Tinamou, which has much higher sequence divergence than the other groups, GC content was higher than the other groups (figs. 6*c* and S3, Supplementary Material online). Coding data sets (C123, C12, and C3) have relatively high GC content and RVC compared with noncoding data sets (CNEE, intron, and UCE) (fig. 6*a* and *b*; see also Figuet et al. 2014). The relatively high GC content in the coding data sets might contribute to the high gCF that supports tree II in C12 with low sequence divergence. Notably, the extent of variation of substitution rates between different nucleotides, such as transition–transversion ratio, appears higher in coding data than in noncoding data (supplementary table S4, Supplementary Material online). C12 also had a greater extent of site rate variation (gamma parameter *α* = 0.02) than the other data sets (*α* = 0.2–1.93) (supplementary table S8, Supplementary Material online).

The high extent of site rate variation might have biased the topologies of phylogenetic trees constructed for loci in C12. Phylogenetic trees were constructed for loci created by sorting the sites of concatenated sequence by estimated site-specific rates (Mayrose et al. 2004) and dividing into the average length of the loci of this data set (table 1) to reduce this bias. Because most sites (92%) were invariant in C12 (supplementary table S6, Supplementary Material online), phylogenetic trees could be estimated for the loci created from ∼10% of sites. In these created loci, gCF that supports tree I became the highest among those for trees I–III (supplementary table S7*a*, Supplementary Material online). Therefore, reducing the extent of site rate variation appears to improve the accuracy of estimating phylogenetic trees in C12. In this analysis, because there were almost no invariant sites in the created loci, the substitution model used was general time-reversible (GTR) + ASC, in which ASC represents ascertainment bias correction of sites (Lewis 2001). The best-fit substitution model estimated for most of the loci was TIM3e + ASC in which transversion rates between some bases (A–C and G–T, and A–T and C–G) and base frequencies are assumed to be equal (supplementary table S7*b*, Supplementary Material online). In the other data sets, because estimated branch lengths at many of the created loci became unusually high (>10) (data not shown), the use of sites sorted by the site-specific rate appeared inappropriate for the other data sets.

These results suggest that tree II was supported often in the high divergence locus sets of intron, C12, and C3 not just due to high sequence divergence but also the complex substitution pattern, which deviates from the substitution model used in this study (GTR + G4), which assumes that nucleotide frequencies and relative substitution rates between different nucleotides stay the same through all branches of the tree (stationary time-homogeneous model), making correction of multiple hits difficult.

## Discussion

In this study, the relationships of the four palaeognath groups (Kiwi, Emu, Rhea, and Tinamou) and the outgroup (ostrich and chicken) were investigated, using three noncoding data sets (CNEE, intron, and UCE) and one protein-coding (CDS) data set. By using different data sets consistently supported Rhea as the group that diverged first among the four groups when the closely related ostrich was used as the outgroup. Together with the result of computer simulation showing that the correct tree topology was supported more often with ostrich than with chicken and both ostrich and chicken as the outgroup, Rhea appears to be the group that diverged first among the four groups. In contrast, the relationship of the four groups (trees I–III, fig. 1*c*) varied depending on the outgroups and data sets used. However, in the whole sequence data of the four groups, the proportion of site pattern (sCF) that supports tree I was about 40% and higher than those supporting trees II and III (about 30%) consistently in all the data sets (table 3). Discordance of the sCF values and the proportion of gene trees (gCF) that supported trees I–III occurred in loci with relatively high sequence divergence of intron and CDS, where gCF was the highest for tree II. High nucleotide composition bias and heterogeneity among the groups in these loci suggested that difficulty of correcting multiple hits due to saturation or the complex substitution pattern, which deviates from the stationary time-homogeneous substitution (SH) model (GTR + G4) used in the construction of phylogenetic trees, was likely cause of the discordance and that tree I is the most likely relationship of the four groups. In high divergence loci of CDS (C3), even sCF supported most highly tree II and the discordance of sCF and gCF also happened in C12 with low sequence divergence. This result suggested that the distortion of the tree topology more likely happens in coding loci than noncoding loci.

### CR1 Retroelement Insertions

Cloutier et al. (2019) analyzed 4,301 informative CR1 retroelement insertions, and Simmons et al. (2022) analyzed 4,345 CR1 insertions, adding 44 loci to the data of Cloutier et al. Both studies supported T7 among the 15 possible tree topologies (T1–T15) of the five palaeognath groups (fig. 1*b*) using multispecies coalescent analyses such as ASTRAL, MP-EST (Liu et al. 2010), and/or SDPquartets (modified SDVquartets; Chifman and Kubatko 2014) (Springer et al. 2020). However, the number of loci that supported the internal branch that separated the group that diverged first (tinamous) from the other three groups (kiwi, emu and cassowary, and rheas) was small: seven in Simmons et al. and five in Cloutier et al. On the other hand, the number of loci that supported the internal branches that separated the group that diverged first from the other three groups for T4 and T1 was six and three, respectively, in Simmons et al. Because the differences were very small, although the bootstrap values that supported the internal branch in T7 were quite high (96% and 95%) by ASTRAL and SDPquartets, Simmons et al. concluded that more retroelement characters are required to resolve the relationships of the palaeognath groups based on a simulation study of their group (Molloy et al. 2021), indicating that there is not sufficient information in the CR1 data.

### Divergence Pattern of the Palaeognaths

**Fig. 7.**
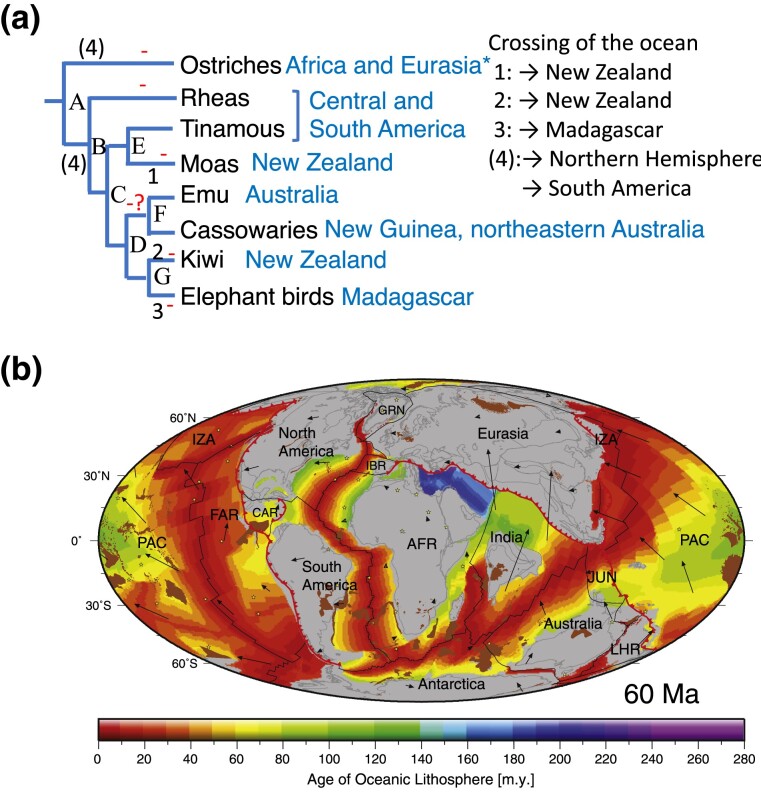
Divergence pattern of palaeognaths and the state of continental drift 60 Ma. (*a*) The divergence pattern of palaeognaths supported by the result of this study. A–G represent ancestors of palaeognath lineages. The numbers 1–3 are crossing of the ocean required by the palaeognath ancestors. The number (4) indicates a likely crossing of the ocean by the ancestor of ostriches or by the ancestor of the common ancestor of the other four groups. Asterisk denotes that fossil records indicated the distribution of ostriches in Africa and Eurasia (Widrig and Field 2022). A dash represents possible flight loss. A question mark indicates a possible loss of flight in the common ancestor of emu and cassowaries. Alternatively flight might be lost independently on the branches leading to emu and cassowaries. (*b*) Reconstruction of global plate 60 Ma. Modified from fig. 18a in Seton et al. (2012). Breakup of Africa and South America: >95 Ma. Breakup of Madagascar from Africa and India: at least 80 Ma. Breakup of New Zealand from Australia: 80 Ma.

Figure 7 *a* shows the divergence pattern of the five palaeognath groups (ostriches, Kiwi [kiwi and elephant birds], Emu [emu and cassowaries], Rhea [rheas], and Tinamou [tinamous and moas]) that the results of this study support together with the results of previous studies. In this pattern, after the divergence between ancestors of ostriches and the other four groups Kiwi/Emu/Rhea/Tinamou (A), divergences between the ancestors of Rhea and Kiwi/Emu/Tinamou (B), Tinamou and Emu/Kiwi (C), and Emu and Kiwi (D) subsequently occurred. Further, the ancestor of Tinamou was divided into those of tinamous and moas (E), Emu into those of emu and cassowaries (F), and Kiwi into those of kiwi and elephant birds (G). In previous molecular studies, the divergence between ostriches and the other four groups (A) was estimated ∼80–50 Ma (80 Ma; Jarvis et al. 2014; Yonezawa et al. 2017, 70 Ma; Grealy et al. 2017, 50 Ma; Prum et al. 2015), and the divergences among the four groups (B–D) (44–38 Ma; Prum et al. 2015, 69–58 Ma; Grealy et al. 2017, 71–67 Ma; Yonezawa et al. 2017). Most of the divergences of the ancestors of Tinamou, Emu, and Kiwi (E–G) (52, 31, and 54 Ma in Grealy et al. 2017; 63, 67, and 62 Ma in Yonezawa et al. 2017) were estimated to have occurred within 10 Myr.

The continental breakup of Africa and South America was completed before 95 Ma (Jacobs et al. 2011; Reguero et al. 2014). Therefore, molecular time estimates suggest that the separation between ancestors of ostriches and the other four groups (A) occurred after the breakup of Africa and South America. Ostriches inhabit or inhabited Africa and Eurasia, and Rhea and Tinamou Central and South America (Widrig and Field 2022). Claramunt and Cracraft (2015) estimated the most common ancestor of Neornithes (Palaeognathae, Galloanserae, and Neoaves) and early ancestors of many groups of Neornithes presented in South America. Therefore, it is likely that the most recent common ancestor of extant palaeognaths also inhabited South America and the ancestor of ostriches moved to Africa and Eurasia, possibly through North America (Claramunt and Cracraft 2015).

However, in Claramunt and Cracraft (2015), the estimated region where the most recent common ancestor of extant palaeognaths inhabited was ambiguous depending on the reconstruction model used. The Palearctic that includes Eurasia and North Africa was a more likely reconstruction than South America when a dispersal–vicariance model was assumed, although South America was estimated as the region when Fitch parsimony was used. Furthermore, recent studies (Mayr 2019; Mayr and Zelenkov 2021) identified that fossils from Central Asia 40 Ma are representatives of extinct groups (Eogruidae and Ergilornithidae) of Struthioniformes (ostriches) and their affinities to extinct groups Palaeotididae and Geranoididae that were spread in Europe and North America in Early Eocene. It should be noted that fossils of ostriches from Africa are only after Miocene 20 Ma (e.g., Leonard et al. 2006; Mikhailov and Zelenkov 2020; Mayr and Zelenkov 2021). Therefore, it is also possible that the most recent common ancestor of extant palaeognaths inhabited Northern Hemisphere and the ancestor of the four groups moved to South America.

After that, the divergences between the ancestors of Rhea and Kiwi/Emu/Tinamou (B), between Tinamou and Emu/Kiwi (C), and between tinamous and moas (F) likely occurred in South America. Then, the common ancestor of Emu and Kiwi and the ancestor of moas moved to the region that includes Australia, New Guinea, and New Zealand, where they mostly inhabit or inhabited. Geological studies indicated that South America, Antarctica, and Australia conformed land that moved in concert after breaking off from Africa (Jacobs et al. 2011) (fig. 7*b*) until the separation of South America and Antarctica and that of Antarctica and Australia completed 50–30 Ma (Teasdale et al. 2003; Reguero et al. 2014). Therefore, the common ancestor of Emu and Kiwi and the ancestor of moas likely moved through Antarctica to their destinations. If it is the case, the ancestor of elephant birds possibly diverged from the ancestor of kiwi in Antarctica, as suggested by Yonezawa et al. (2017).

In this scenario, the ancestors of the palaeognath groups had to cross the ocean at least three times: to New Zealand, which had already separated from Australia 80 Ma (Gurnis and Hall 2004) by the ancestors of kiwi (1) and moas (2), and to Madagascar that broke off from Africa and India and became an island at least 80 Ma (Seton et al. 2012) by the ancestor of elephant birds (3). It is also likely that the ancestor of ostriches moved from South America to Northern Hemisphere or the ancestor of the other four groups moved to South America crossing the ocean. It indicates that at least the ancestors on the four peripheral branches (to moas, kiwi, elephant birds, and flighted tinamous) and the ancestor of ostriches or the common ancestor of the four groups had to fly (fig. 7*a*). Therefore, it is likely that the ancestors on the stem branches of the palaeognath divergences, including the common ancestor of all the five groups, maintained the ability to fly, and loss of flight occurred independently in most of the peripheral branches including that of ostriches (e.g., Phillips et al. 2010; Haddrath and Baker 2012; Baker et al. 2014; Mitchell et al. 2014; Grealy et al. 2017; Cloutier et al. 2019; Sackton et al. 2019; Widrig and Field 2022). Then, as Grealy et al. (2017) suggested, loss of flight occurred six times (fig. 7*a*).

### Discordance of gCF and sCF and the Complex Substitution Pattern

Previously, it was shown that a higher extent of gene tree error was associated with higher GC content for nuclear genes of mammals (Romiguier et al. 2013; Scornavacca and Galier 2017) and bees (Romiguier et al. 2016; Bossert et al. 2017). It was attributed to high recombination rate in high GC content regions, due to GC-biased gene conversion at meiotic recombination (Galtier et al. 2001) in some studies (Romiguier et al. 2013, 2016). However, in this study, the high GC bias/heterogeneity and sequence divergence were associated with an increase of support for tree II, not with random support of different tree topologies, and in the data sets used, the loci likely subjected to recombination among the palaeognath groups and outgroups were excluded. The extent of association of GC-biased substitution with high recombination appears to vary depending on the species. In Capra and Pollard (2011), the association was positive in mammalian species and fruit flies, but negative in chicken, although a positive association of recombination rate and GC content was observed in chicken (Webster et al. 2006). Therefore, rather than a high recombination rate, difficulty in correcting multiple hits due to the complex substitution pattern would be more likely cause of the high tree II support in loci with high GC bias/heterogeneity and sequence divergence, as was shown for mitochondrial sequence of palaeognaths (Phillips et al. 2010; Grealy et al. 2017) and mammals (Phillips and Pratt 2008) and nuclear genes of birds (Nabholz et al. 2011).

High support for tree II by sCF, which is based on parsimony, occurred in some of high sequence divergence loci of coding data (C123 and C3). However, the support for tree II by gCF was higher than by sCF. Parsimony is generally thought to be more subjected to distortion of tree topology due to the effect of multiple hits than model-based methods such as the maximum likelihood method used in this study (e.g., Kuhner and Felsenstein 1994; Philippe et al. 2005, 2017). The substitution model (GTR + G4) used in this study is a SH model that assumes equilibrium nucleotide frequencies and constancy of substitution rates between different nucleotides. Therefore, as high GC bias and heterogeneity observed in these loci indicated, deviation from the SH model might be a cause of the higher extent of distortion of tree topology by the maximum likelihood method than the parsimony-based method.

### Estimation Error of Gene Trees

Because of the limited sequence lengths, gene trees are prone to estimation error. Particularly, in CNEE and C12 which have lower sequence divergence than the other data sets (fig. 3), the number of branches with length zero in a gene tree was much higher (8.6 for CNEE and 5.16 for C12 on average) than the other data sets (2.2 or lower) (tables 4 and S9, Supplementary Material online). It indicates that a small amount of phylogenetic signal is contained in individual loci of these data sets, particularly those of CNEE with the short sequence length (tables 1 and S1, Supplementary Material online).

**Table 4:** Number of Internal Branches With Zero Length and Topological Difference of Gene Trees Constructed by IQTREE and RAxML

Data Set	Number of Zero-Length Branches		*P*_DIF_ (%)	*D*_RF_	*r*	Gene Tree–Based Approach			
	All	Internal Branch				IQ-TREE		RAxML	
						Tree	BP	Tree	BP
CNEE	8.64 ± 3.00	3.41 ± 1.60	72.57	2.96 ± 2.74	0.60	I	25.1	III	34.9
Intron	1.15 ± 1.47	0.21 ± 0.50	6.77	0.16 ± 0.61	0.59	III	32.9	III	32.9
UCE	0.79 ± 1.00	0.12 ± 0.35	4.70	0.10 ± 0.52	0.49	III	30.5	III	29.2
C123	1.55 ± 1.40	0.27 ± 0.51	9.62	0.21 ± 0.70	0.50	II	31.2	II	32.1
C12	5.16 ± 3.76	1.57 ± 1.69	35.11	1.18 ± 2.12	0.62	I	27.4	I	27.3
C3	2.24 ± 1.61	0.44 ± 0.64	14.50	0.33 ± 0.88	0.48	I	29.8	II	31.4

Note.—Sequence data of four groups (K, E, R, and T) were used. In counting the number of branches with zero length, branch length 10^−6^ was regarded as zero. *r* was all significant with 10^−6^ level.

*D*_RF_, Robinson–Foulds distance between topologies of gene trees constructed by IQ-TREE and RAxML; *r*, Pearson’s correlation coefficient between the number of branches with zero length and the topological distance (*D*_RF_) between gene trees constructed by IQ-TREE and RAxML; *P*_DIF_, percentage of loci in which topologies of gene trees constructed with IQ-TREE and RAxML differed.

The presence of zero-length branches can cause the topological difference in trees constructed using different software. In the results shown above, phylogenetic trees were constructed using IQ-TREE (version 2.1.3, Minh et al. 2020a). However, when RAxML (version 8.2.12, Stamatakis 2014) was used, the topologies of gene trees were different from those constructed using IQ-TREE in some of the loci (5–73% in different data sets), and the extent of topological difference (*D*_RF_) was positively correlated with the number of zero-length branches (Pearson's correlation coefficient [*r*] between *D*_RF_ and the number of zero-length internal branches was 0.5–0.6; table 4). When there is a zero-length internal branch, sequence data do not contain phylogenetic signal regarding the formation of clades that the internal branch divides. Therefore, it appears that the topological difference between gene trees constructed using IQ-TREE and RAxML occurred because of the difference in their algorithms for constructing a bifurcating tree. This topological difference of estimated gene trees can affect the gCF values and trees constructed by the gene tree–based approach. For example, in CNEE, gCF that supports tree I was the highest when IQ-TREE was used (table 3). In contrast, gCF that supports tree III became the highest when RAxML was used (tables 5 and S10, Supplementary Material online). However, when loci in which estimated lengths of internal branches of all the three possible trees of the four groups were zero were excluded, gCF that supports tree I became the highest even when RAxML was used (tables 5 and S11, Supplementary Material online). These results indicate that particularly in the loci with low sequence divergence and/or short sequence length, constructed gene trees likely contain many zero-length branches and that it could be associated with topological error of constructed gene trees.

**Table 5:** gCFs That Support Trees I–III When Gene Trees Were Constructed by IQ-TREE and RAxML in CNEE and C12

Loci Used	Tree	CNEE		C12	
		IQ-TREE	RAxML	IQ-TREE	RAxML
All	I	26.7	20.7	29.4	27.6
	II	24.6	18.2	28.0	27.5
	III	23.0	34.9	26.0	28.3
	Total	74.3	73.7	83.5	83.4
	No. of loci used	12,561		5,374	
Loci with zero-length internal branches excluded	I	30.3	29.2	32.2	31.9
	II	29.9	28.9	31.2	31.3
	III	27.2	28.4	28.1	28.2
	Total	74.3	86.4	74.3	74.3
	No. loci used	5,205		3,961	

Branch lengths were estimated fixing the tree topologies to trees I–III in which the branching patterns within the four palaeognath groups were those in the trees constructed from concatenated sequences. Branch length 10–6 or smaller was regarded as zero.

Tree topologies constructed by the gene tree–based approach using gene trees constructed by IQ-TREE and RAxML were also different in some data sets (CNEE and C3) (table 4). In addition, the constructed tree topologies also varied depending on the data sets used (supplementary table S12, Supplementary Material online). Therefore, they were likely affected by factors other than zero-length branches such as the bias in estimated tree topologies observed in loci with high sequence divergence, nucleotide composition bias/heterogeneity, and the number of loci used, which are much smaller in intron (1,400 loci) and UCE (1,800 loci) than the other data sets (5,400 loci for coding data and 12,600 loci for CNEE).

### Relationship of the Four Groups and Gene Tree Discordance

Poor phylogenetic signal that individual loci likely contain can be avoided by using concatenated sequence. However, a high extent of gene tree discordance was indicated by a similar extent of support (about 30% or higher) for all the three possible relationships of the four species groups (trees I–III) by the site pattern (sCF) decisive for the three relationships (trees I–III) and the branching pattern of gene trees (gCF) in all the data sets (table 3). In such a case, it is inappropriate to construct a phylogenetic tree from concatenated sequence assuming the same tree topology in all the loci and discordance among gene trees should be accounted. Moreover, it is known that there is a case called anomaly zone in which even a phylogenetic relationship at a locus (gene tree without estimation error) discordant with the species tree has a higher probability of occurrence than a gene tree concordant with the species tree (Degnan and Rosenberg 2009; Warnow 2015; Edwards et al. 2016). However, regarding a rooted tree of three species or unrooted tree of four species (see trees I–III in fig. 1*c*), even in the presence of ILS under which tree topologies can be different from locus to locus due to ancestral polymorphism, the coalescent theory predicts that a gene tree concordant with the species tree always has the highest probability of occurrence (1–2/3e^−*T/*Ne^) and the probability of occurrence for each of the other two trees is 1/3e^−*T/*Ne^, where *T* is the number of generations of the internal branch of the species tree and Ne is the effective population size (Pamilo and Nei 1988; Bryant and Hahn 2020). If the grouping of Kiwi, Emu, Rhea, and Tinamou is considered solid, the phylogenetic tree of the four groups can be regarded as the species tree of ancestral populations of these groups and the most highly supported tree I is the most likely relationship of the four groups.

Applying the frequency of tree I as 40% to the probability of occurrence 1–2/3e^−*T/*Ne^ predicted for the gene tree concordant with the species tree, *T*/Ne becomes approximately 0.11. Assuming that the ancestral population size was 10,000–100,000 (kiwi from present to 120,000 years ago, Weir et al. 2016; current population size in the order of 10^4^ for kiwi and cassowaries and 10^5^ for emu and tinamous in BirdLife database; BirdLife International 2022) and the generation time was 3 years (median for bird species 2.9 years, Bird et al. 2020; female maturation time: 1–3 years for palaeognath species in AnAge database; Tacucu et al. 2013), *T* becomes 11,000–110,000 generations, corresponding to 33,000–330,000 years. This value is much smaller than previous molecular time estimates (2–4 Myr: 2 Myr; Prum et al. 2015 [tree II]; 2 Myr; Grealy et al. 2017 [tree I], 3 Myr; Yonezawa et al. 2017 [tree I]) based on the estimated branch lengths and fossil calibrations. It should be noted, however, that the estimated value of *T* is subjected to large error because it is not known how well the generation time and the population size used are applied to the ancestral populations and what influence natural selection would have on the estimate because the coalescent theory assumes the neutrality of alleles.

### Loci and Species to Be Used

This study showed that loci with high sequence divergence and nucleotide composition bias/heterogeneity are likely to have distorted tree topology due to saturation or deviation from the SH substitution model used in tree construction. In addition, tree topology can be more likely to be distorted with distantly related outgroup than closely related outgroup. Therefore, it is important to examine the relevancy of loci and species used to resolve the phylogenetic relationship in question.

In this study, the comparison of tree support by site pattern and branching pattern of gene trees in loci with different divergence levels revealed distortion of tree topology because of difficulty in correcting multiple hits due to saturation or deviation from the substitution model assumed. Just looking at the sequence divergence level of loci, saturation does not appear to occur in the data sets used. Even in top 10% high divergence locus sets, the longest sum of branch lengths between the palaeognath groups was smaller than 1 and 0.5 with or without the outgroups (supplementary table S13, Supplementary Material online), and in the phylogenetic informativeness (PI) approach, which provides theoretical prediction of the resolving power of branching pattern with respect to the sequence divergence (Townsend 2007; López-Gir^á^ldez and Townsend 2011), the peaks of the resolving power were outside of the common ancestral node of the four groups (supplementary fig. S4, Supplementary Material online).

The problem that of compositional heterogeneity and/or deviation from the SH model creates bias in estimating tree topology was recognized in previous studies. The methods that relax the assumptions of the SH models such as the stationarity along the branches (e.g., Lake 1994; Lockhart et al. 1994; Steel et al. 1995; Yang and Roberts 1995; Galtier and Gouy 1995, 1998; Gu and Li 1996, 1998; Tamura and Kumar 2002; Foster 2004; Blanquart and Lartillot 2006), constancy of site-specific rates (Lartillot and Philippe 2004; Wu and Susko 2009), and both (Gowri-Shanker and Rattray 2006; Blanquart and Lartillot 2008; Jayaswal et al. 2014) have been developed. However, these substitution models were rarely used because they are computationally demanding and not easy to use (Betancur-R et al. 2013; Naser-Khdour et al. 2019). It should also be noted that each of these models considers relaxing some of the assumptions of the SH models (Gowri-Shanker and Rattray 2006), and the effectiveness of these models for obtaining correct tree topologies is not well investigated (Betancur-R et al. 2013). Furthermore, in the presence of ILS, although the construction of gene trees is necessary, the estimation error is likely to occur using the complex models due to the high number of parameters to be estimated (Ho and Jermiin 2004).

In addition, although tests or measures of the extent of violations of the assumptions of the SH models have been developed (Rzhetsky and Nei 1995; Steel et al. 1995; Kumar and Gadagkar 2001; Weiss and von Haeseler 2003; Jayaswal et al. 2011; Wu and Susko 2011; Dutheil et al. 2012; Brown and Thompson 2017; Duchêne et al. 2017; Jermiin et al. 2017), the extent of violations that causes bias for the estimated tree topology is not clear. Even a small extent of nucleotide composition heterogeneity can distort estimated tree topologies using an SH model if the internal branch is short (Conant and Lewis 2001; Jermiin et al. 2004). The lengths of the internal branch of the four palaeognath groups were in the order of 10^−3^ on average, which are 5–20% of external branches in the data sets used in this study (supplementary table S14, Supplementary Material online). To see the performance of the tests, matched-pair tests (tests of symmetry, marginal symmetry, and internal symmetry) for violations of the SH assumptions (Ababneh et al. 2006; Jermiin et al. 2017; Naser-Khdour et al. 2019), which are available in IQ-TREE, were applied to the data sets used in this study. The number of loci in which all the pairs of sequences passed all three tests was small: 20–30% of loci of CNEE and C12 with low divergence and only a small number of loci for intron, UCE, and C3 (5, 0, and 115) with high divergence (supplementary table S15, Supplementary Material online). It should also be noted that the performance of the three tests varied; the number of loci that significantly violated the SH assumptions was much smaller for the test of internal symmetry than those for the tests of symmetry and marginal symmetry: 60–70% for CNEE and C12 and 10% or lower for intron, UCE, and C3.

These results indicate that although the tests for violations of the assumptions are available, it is not easy to use them for choosing loci appropriate for constructing phylogenetic trees. At this point, a practical way could be to use RY-coding or loci with a low extent of divergence and nucleotide heterogeneity to mitigate the effect of saturation and deviation from the SH models, focusing on the resolution of the relationships at a particular node. However, it may cause a loss of information in sequence data and increase the extent of gene tree estimation error. Therefore, an effective way of choosing appropriate loci and species such as outgroups to resolve the phylogenetic relationships taking into account the complexity of substitution patterns is necessary for phylogenomic study.

## Materials and Methods

### Sequence Data Used

Sequence data of Cloutier et al. (2019) and Sackton et al. (2019) were downloaded from Dryad Digital Repository. Noncoding sequences of 12,676 CNEEs, 5,016 introns, and 3,158 UCEs from Cloutier et al. (2019) included species from the five groups of extant palaeognaths, Apterygiformes (*Apteryx haastii* [great spotted kiwi], *Apteryx owenii* [little spotted kiwi], *Apteryx rowi* [Okarito brown kiwi], and *Apteryx mantelli* [North Island brown kiwi]); Casuariiformes (*Casuarius casuarius* [southern cassowary] and *Dromaius novaehollandiae* [emu]); Rheiformes (*Rhea americana* [greater rhea] and *Rhea pennata* [lesser rhea]); Tinamiformes (*Eudromia elegans* [elegant crested-tinamou], *Tinamus guttatus* [white-throated tinamou], *Nothoprocta perdicaria* [Chilean tinamou], and *Crypturellus cinnamomeus* [thicket tinamou]); Struthioniformes (*Struthio camelus* [ostrich]); extinct Dinornithiformes (*Anomalopteryx didiformis* [little bush moa]); and chicken (*Gallus gallus*).

In the following analyses, the moa was regarded as in the group of tinamous, and ostrich and chicken as the outgroup. Branch lengths were estimated for each locus fixing the tree topology constructed for concatenated sequences. When branch lengths of some species from the common ancestral node of palaeognaths excluding the outgroups (ostrich as well as chicken) (*N*_CA_; see fig. 1*a*) relative to those of the other species were more than five times longer than those of concatenated sequences, the loci were excluded for introns and UCEs because in such cases, the values of branch lengths were large (>1 substitution per site) (75 intron loci and 174 UCE loci). As pointed out by Simmons et al. (2022), it is likely that UCE loci contain sequences that are not orthologous. No such excluded loci existed for CNEEs because of the low divergence level (supplementary table S1, Supplementary Material online).

In Sackton et al.'s (2019) expanded data of CDSs, there were 47 species in total: 13 palaeognaths in which *A. mantelli* was missing out of 14 species in Cloutier et al. and 31 neognaths, alligator, anole lizard, and turtle. Out of 11,271 loci, sequences of palaeognaths and chicken were extracted for 8,721 loci that contained all five groups of palaeognaths and both ostrich and chicken. When there were multiple sequences of the same species, they were combined into one sequence. It should be noted that they were nonoverlapping in all the cases. Similar to the introns and UCEs, loci in which the relative branch length of a species to the other species was five times longer than those of concatenated sequences were excluded. Because there were high variations in branch lengths among species in the CDS data, using more stringent criteria than for Cloutier et al.'s data, loci in which there were long branches to species from the common ancestral node of palaeognaths excluding the outgroup ostrich (*N*_CA_; see fig. 1*a*) (>1) and the branch length of a species was five times longer than those of other species in the same group were also excluded (1,102 loci excluded and 7,542 loci left). The likelihood ratio test was carried out for site models M1a (nearly neutral) versus M2a (positive selection) and M8 (positive selection) versus M8a (dN/dS (*ω*) = 1) by using codeml of PAML 4.9j (Yang 2007) to detect positively selected loci. When at least either of the results of the likelihood ratio test and the Bayes empirical Bayes (BEB) method was significant at 1% level, the locus was excluded (705 loci excluded and 6,837 loci left). At the loci with the significant results, the substitution rates were 20–30% higher than the loci with nonsignificant results and the number of sites was on average 27% longer. These properties apparently contributed to a higher power of the tests at these loci. In protein-coding regions, it is known that the divergence level of third codon positions is much higher than that of the first and second positions because substitutions at the former are mostly synonymous and those at the latter are nonsynonymous. Therefore, the CDSs were divided into first and second codon positions (C12) and third positions (C3) and analyzed separately in addition to the data including all codon positions (C123).

Recombination was detected by using 3SEQ version 1.7 build 170612 (Lam et al. 2018). Loci in which recombination was detected between any pair of the five groups of palaeognaths and chicken were excluded (Norecomb sets) from the data sets described above from which loci with long relative or actual branch lengths and positive selection detected were excluded (All sets) (supplementary table S1, Supplementary Material online). Whereas the number of loci was slightly reduced for CNEEs (from 12,676 to 12,561) and CDSs (from 6,837 to 5,374), more than 50% of loci were excluded for introns (from 4,941 to 1,802) and UCEs (from 2,982 to 1,363) (supplementary table S1, Supplementary Material online). The sequence divergence of the remaining loci decreased 1% to 15% (1%, 5%, 15%, and 5% for CNEEs, introns, UCEs, and CDSs, respectively; supplementary table S2, Supplementary Material online). However, because the results essentially remained the same (see supplementary table S16, Supplementary Material online), the results for the Norecomb sets were shown.

### Phylogenetic Analyses

Phylogenetic trees were constructed by using IQ-TREE 2.1.3 (Minh et al. 2020a) with the substitution models, GTR model + G4 (assuming the gamma distribution with four discrete categories for rate across sites), and models in GHOST method (GTR + H4, GTR*H4, and GTR + FO*H4) (Crotty et al. 2020). Using the models in the GHOST method, the likelihood values of the estimated trees became higher than those with GTR + G4. However, the effect on tree topologies constructed appeared small (supplementary table S16, Supplementary Material online). Therefore, the results using GTR + G4 were shown.

In the partitioned approach, sequence was partitioned by each locus and GTR + G4 was assumed. -Q (each partition has its own set of branch lengths) and -p option (branch lengths of partitions are proportionally scaled) (Chernomor et al. 2016) were used. RAxML 8.2.12 (Stamatakis 2014) was also used with GTR + G4. Bootstrap probabilities were computed using the ultrafast bootstrap (UFBoot) method (Hoang et al. 2018) with 1,000 replications. In the gene tree–based approach, species trees were estimated by ASTRAL 5.7.4 (Zhang et al. 2018). The bootstrap test was performed with replications of the number of loci included in the data.

In the preliminary analysis, AU test (Shimodaira 2002) for the 15 possible tree topologies of the five lineages (fig. 1*b*) was carried out with 10,000 replications. The UFBoot values were similar to or more conservative than the significance value of the AU test.

### Computer Simulation

Sequence data were simulated by Seq-gen 1.3.4 (Rambaut and Grass 1997), assuming substitution model GTR + G. Estimated branch lengths, base frequencies, the extent of rate variation across sites (*α*), and the substitution rate parameters for concatenated sequences of different data sets for the 15 possible tree topologies of the five groups of palaeognaths (fig. 1*b*) assuming GTR + G8 were used for generation of sequence data. From generated sequences of all the species (15 species for noncoding and 14 species for coding data sets), sequences with different combinations of outgroups were extracted. Phylogenetic trees were constructed using IQ-TREE 2.1.3 assuming GTR + G4. Sequence length (*L*) was set to 300, 600, 900, and 1,800. A total of 10,000 replications were done for the noncoding and 5,000 replications for the protein-coding data sets when *L* was 300. For the other sequence lengths, 1,000 replications were done. *D*_RF_ (Robinson and Foulds 1981) between the tree topology assumed to generate the sequence data (model tree) and that constructed from the sequences was computed by using RF.dist in phangorn package of R language (Schliep 2011).

### Phylogenetic Informativeness

Profile of PI (Townsend 2007) was obtained by using PhyInformR (Dornburg et al. 2016). Site-specific rates of substitution (Mayrose et al. 2004) of different data sets were computed by IQ-TREE 2.1.3 (Minh et al. 2020a), assuming GTR + G4 for the constructed tree from the concatenated sequence. PI values were shown relative to the rooted tree of the CNEE set whose branch lengths were estimated under the assumption of constant rate using PAML 4.9j (Yang 2007). Site rates were multiplied by the ratio of the total branch length of the tree constructed for the concatenated sequence of the data set to that for the CNEE (Moeller and Townsend 2011; Dornburg et al. 2017). Because *A. mantelli* was missing in the CDS data, the external branch length to this species was excluded in the calculation of total branch lengths of the noncoding sequence data sets.

### Relative Compositional Variability

RCV (Phillips and Pratt 2008) was computed as follows to examine heterogeneity of nucleotide composition among species groups:


$$\mathrm{RCV}=\overset{n}{\underset{i}{\sum }}\;(\left|A_{i}-A^{*}|+|T_{i}-T^{*}|+|C_{i}-C^{*}|+|G_{i}-G^{*}\right|)/nt$$

where *A_i_*, *T_i_*, *C_i_*, and *G_i_* are the average frequencies of each nucleotide of the species group, A*, T*, C*, and G* are averages of all the species groups, *n* is the number of species groups, and *t* is the number of sites. RCV was computed for only sites with no missing or ambiguous data.

### Concordance Factors

gCFs and sCFs (Minh et al. 2020b) were computed for three possible unrooted trees of four palaeognath groups (kiwi, emu and cassowary, rheas, and tinamous and moa) (trees I–III, fig. 1*c*) in which the branching patterns within the groups were fixed as those in the trees constructed for concatenated sequences using concatenated sequences and sequences of individual loci. Gene trees were constructed with GTR + G4. gCFs and sCFs that support the internal branches of trees I–III were extracted from the output. IQ-TREE 2.1.3 (Minh et al. 2020a) was used to compute gCFs and sCFs. For computation of sCFs, the number of quartets of sequences randomly sampled around each internal branch was set to 5,000. In sequence data used to compute gCFs and sCFs, outgroups were excluded.

## Supplementary Material

evad092_Supplementary_Data

## Data Availability

The sequence data used in this study are available at the Dryad Digital Repository: https://doi.org/10.5061/dryad.1jwstqjzs.
