# Supplementary material for: Effect of Different Types of Sequence Data on Palaeognath Phylogeny
Source: Genome Biol Evol. 2023 May 25;15(6):evad092. doi: 10.1093/gbe/evad092 (PMC10262969; doi:10.1093/gbe/evad092)
Supplement: evad092_Supplementary_Data [file evad092_supplementary_data.zip › figures_sup.pdf]

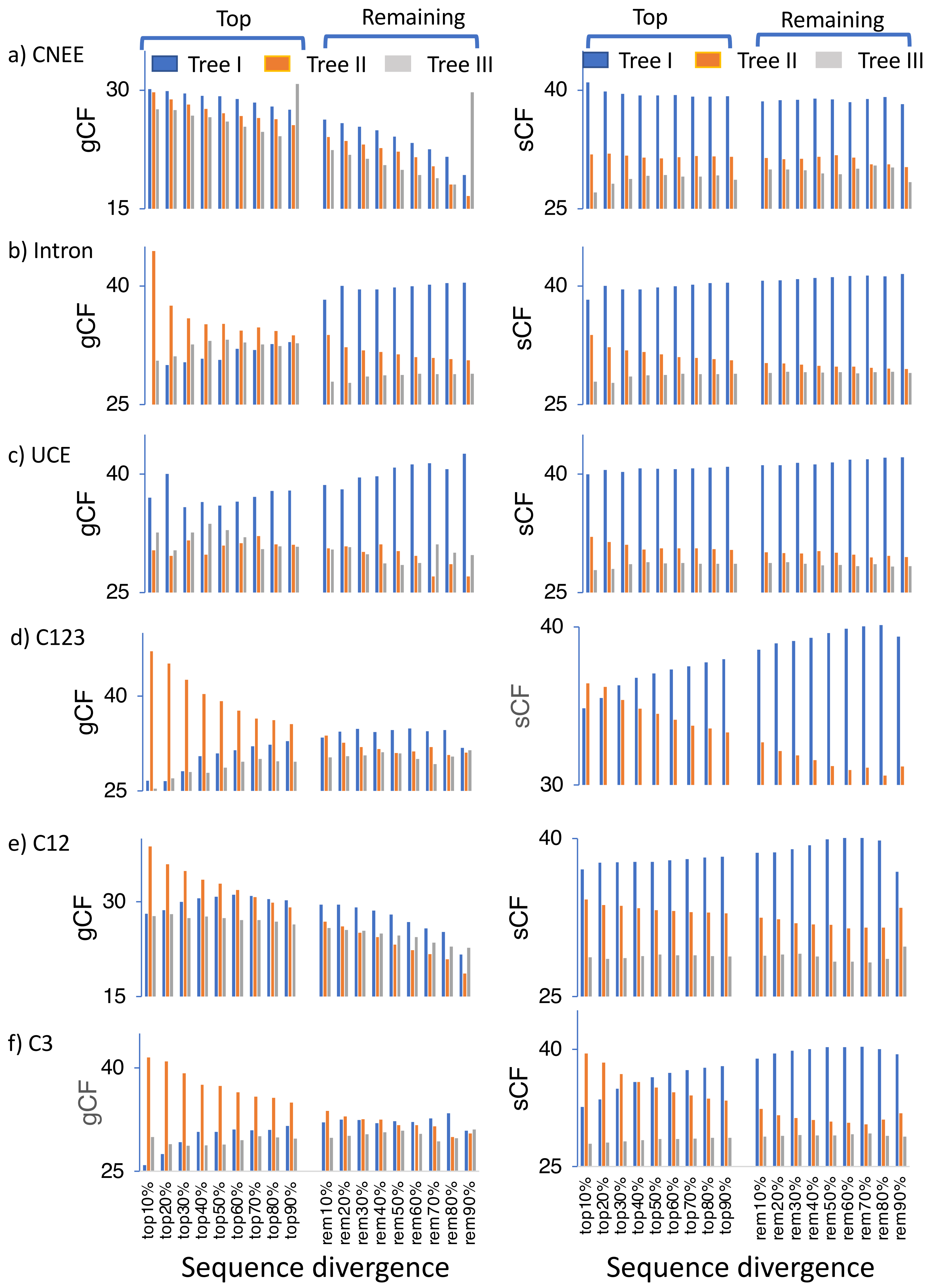

Figure S1. gCFs and sCFs supporting tree I - III of the four palaeognath groups for top- and rem-10% to 90% locus-sets of different sequence divergence levels.

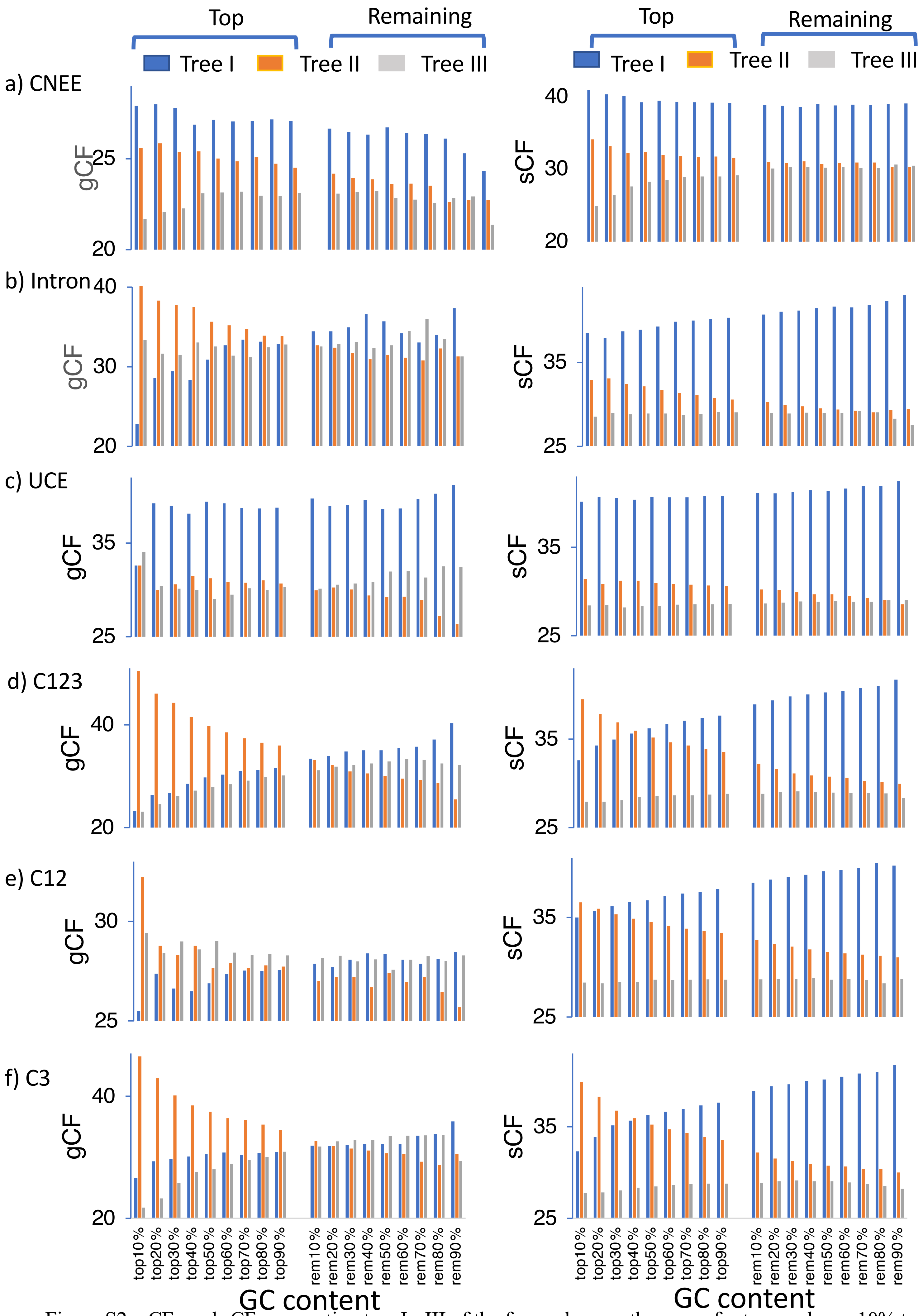

Figure S2. gCFs and sCFs supporting tree I - III of the four palaeognath groups for top- and rem-10% to 90% locus-sets of different levels of GC content.

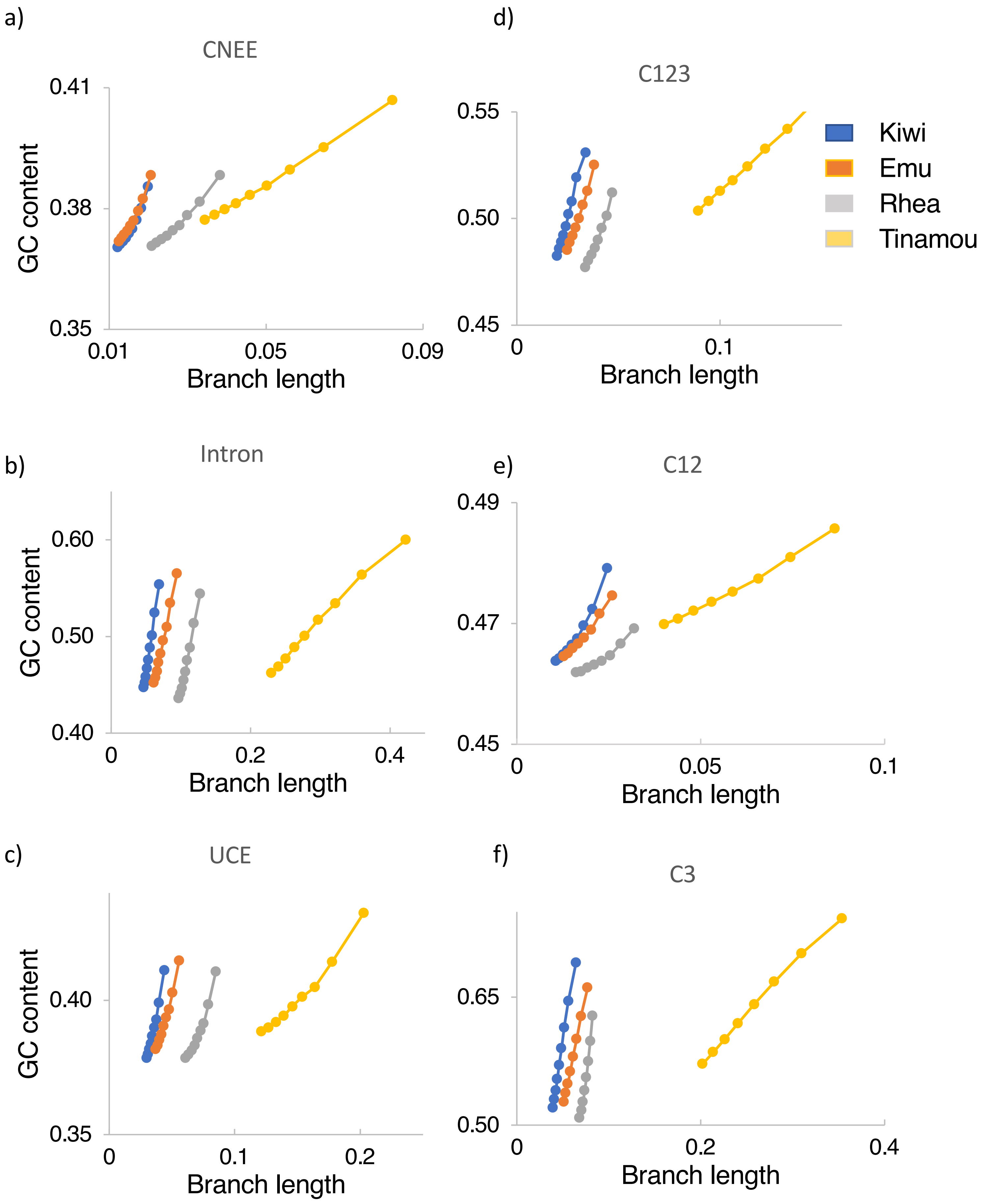

Figure S3. Relationship between GC content and sequence divergence of the four palaeognath groups.

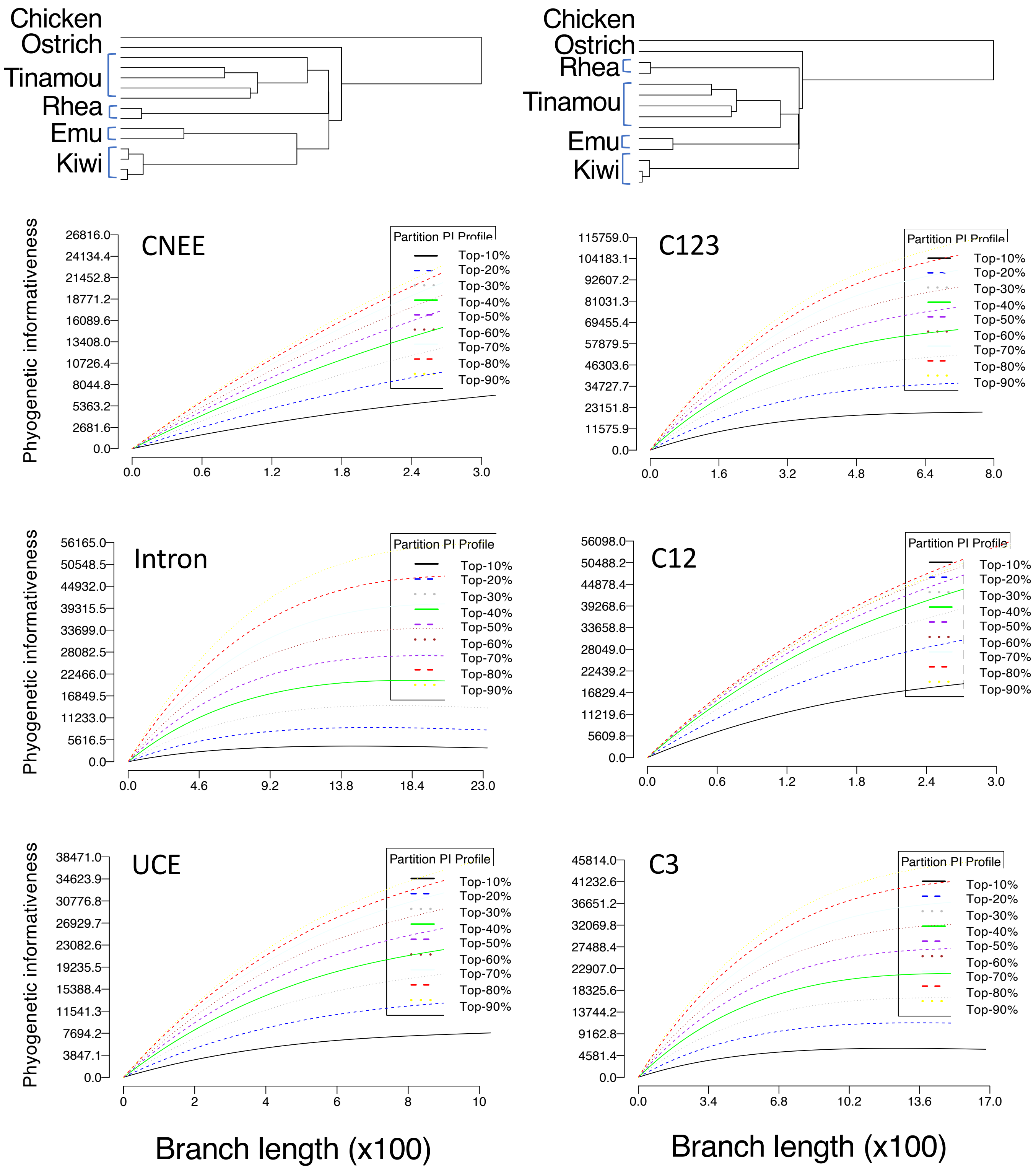

Figure S4. Phylogenetic informative profile of top-10% to top-90% locus sets of different datasets.
